# Highly accurate response prediction in high-risk early breast cancer patients using a biophysical simulation platform

**DOI:** 10.1007/s10549-022-06722-0

**Published:** 2022-09-05

**Authors:** Frederick M. Howard, Gong He, Joseph R. Peterson, J. R. Pfeiffer, Emmy Earnest, Alexander T. Pearson, Hiroyuki Abe, John A. Cole, Rita Nanda

**Affiliations:** 1 Department of Medicine, University of Chicago, 5841 S. Maryland Avenue | MC 2115, Chicago, IL 60637 USA; 2 SimBioSys, Chicago, IL USA; 3 Department of Radiology, University of Chicago, Chicago, IL USA

**Keywords:** Neoadjuvant chemotherapy, MRI, Simulation, Pathologic complete response, Prognostic Biomarker

## Abstract

**Purpose:**

Pathologic complete response (pCR) to neoadjuvant chemotherapy (NAC) in early breast cancer (EBC) is largely dependent on breast cancer subtype, but no clinical-grade model exists to predict response and guide selection of treatment. A biophysical simulation of response to NAC has the potential to address this unmet need.

**Methods:**

We conducted a retrospective evaluation of a biophysical simulation model as a predictor of pCR. Patients who received standard NAC at the University of Chicago for EBC between January 1st, 2010 and March 31st, 2020 were included. Response was predicted using baseline breast MRI, clinicopathologic features, and treatment regimen by investigators who were blinded to patient outcomes.

**Results:**

A total of 144 tumors from 141 patients were included; 59 were triple-negative, 49 HER2-positive, and 36 hormone-receptor positive/HER2 negative. Lymph node disease was present in half of patients, and most were treated with an anthracycline-based regimen (58.3%). Sensitivity and specificity of the biophysical simulation for pCR were 88.0% (95% confidence interval [CI] 75.7 – 95.5) and 89.4% (95% CI 81.3 – 94.8), respectively, with robust results regardless of subtype. In patients with predicted pCR, 5-year event-free survival was 98%, versus 79% with predicted residual disease (log-rank *p* = 0.01, HR 4.57, 95% CI 1.36 – 15.34). At a median follow-up of 5.4 years, no patients with predicted pCR experienced disease recurrence.

**Conclusion:**

A biophysical simulation model accurately predicts pCR and long-term outcomes from baseline MRI and clinical data, and is a promising tool to guide escalation/de-escalation of NAC.

**Supplementary Information:**

The online version contains supplementary material available at 10.1007/s10549-022-06722-0.

## Background

Neoadjuvant chemotherapy (NAC) is the cornerstone of treatment for high-risk, early breast cancer (EBC), facilitating breast-conserving surgery and allowing for an in vivo assessment of sensitivity to treatment [1]. It has been repeatedly demonstrated that response to NAC is a strong predictor of long-term outcomes. Those who achieve a pathologic complete response (pCR) have the best event-free survival (EFS) [2, 3], although pCR rates vary among cancer subtypes. A number of emerging treatment strategies have demonstrated further improvements in pCR rates, including carboplatin in triple-negative breast cancer (TNBC) [4], and immunotherapy in high-risk hormone receptor positive (HR +) disease [5] and TNBC [6, 7]. The administration of intensified neoadjuvant therapy comes with an increase in toxicities—in the case of carboplatin the side-effects are usually short term, but immunotherapy can cause irreversible and life-altering endocrinopathies. Therefore, tools which can accurately predict pCR to specific regimens that allow for treatment optimization and potentially minimize toxicities could lead to the realization of precision medicine.

Several biomarkers have been identified to predict response to neoadjuvant chemotherapy. In addition to molecular features, a number of pre-treatment radiomic characteristics are associated with treatment response, including simple morphologic features like tumor shape [8] and sphericity [9]. Various radiomics [10]studies support the correlation of radiomic features with underlying disease biology and treatment sensitivity [11, 12]. Other studies have shown that the combination of radiomic features with clinical data-enhanced response prediction [9, 13, 14]. These studies, however, require robust validation, and thus, have not yet made their way into routine clinical practice.

Multiscale cancer modeling allows for dynamic simulation of tumor response on both a spatial and temporal scale [15]. TumorScope (SimBioSys, Chicago, IL) is a commercial multiscale biophysical simulation platform that utilizes pre-treatment patient information and pre-treatment MRI to model tumor response over time. The platform accounts for the individual physical and biological traits of each cancer and has previously been used for the in silico simulation of cancer response on a per-patient basis [16]. We present a retrospective, independent, blinded evaluation of this biophysical simulation model as a tool to predict pCR to a variety of different NAC regimens in breast cancer.

## Methods

### Study design and population

We conducted a retrospective single-center evaluation of a biophysical simulation model for the prediction of pCR. The study included patients age 18 or older who received NAC at the University of Chicago for EBC between January 1st 2010 and March 31st 2020 under an institutional review board approved protocol. For inclusion in the study, patients had to be diagnosed with invasive breast cancer and all subtypes and histology of breast cancer were accepted, and had a pre-treatment dynamic contrast enhanced (DCE) MRI available. There was no restriction on magnet strength (1.5 T or 3 T) or machine manufacturer, and MRI series required for inclusion were a pre-contrast T1-weighted image, and images nearest to 200 s–300 s (early post-contrast) and 500 s–600 s (late post-contrast) after contrast administration. Patients with bilateral breast tumors were also eligible and tumors were independently modeled. Patients receiving experimental regimens, those with metastatic disease, and patients receiving neoadjuvant endocrine therapy alone were excluded. Given an a priori estimate of model specificity of 80% for pCR, compared to average pCR rates of approximately 19% in the general population [2], a sample size of 17 is required for each group analyzed to maintain a power of 95% at the two-sided alpha = 0.05 significance level.

Pre-treatment clinical data and MRI were analyzed using the biophysical simulation model for prediction of response by investigators who were blinded to actual response data. The primary outcome was sensitivity and specificity of the biophysical simulation model for prediction of pCR, defined as absence of invasive carcinoma in the breast or lymph nodes at the time of surgery (ypT0/Tis ypN0). Secondary end points included prediction of tumor volume over time and association of predicted pCR with EFS and overall survival (OS). Subgroup analysis was planned for breast cancer subtype and chemotherapy regimen. The analysis follows the REMARK guidelines for reporting on tumor prognostic markers [17].

### Model design

**Fig. 1:**
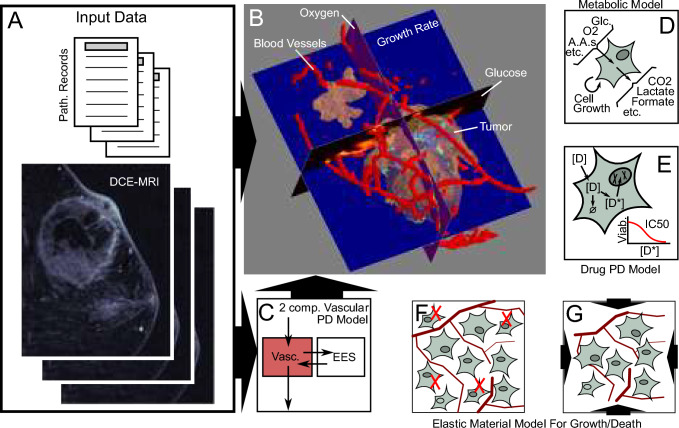
An Overview of the Biophysical Simulation Model. **A** Patient imaging and pathology data are used as inputs in order to construct a comprehensive 3D virtual tumor for simulation. **B** Imaging data are segmented into tissues of interest, including the tumor (tan), blood vessels (red), as well as surrounding healthy adipose and fibroglandular tissues (not shown for clarity). **C** DCE-MRIs are further used to fit a two compartment pharmacodynamic model of tissue perfusion using a modified Tofts model [20]. This model is used within the simulations to provide nutrients and drugs to the simulated tissues, as well as to clear away any byproducts of the tissue's metabolism. **D** During the simulations, proprietary genome-scale metabolic models are used to predict the metabolic behaviors of the cells within each voxel [21]. This includes the rates at which cells utilize resources (such as glucose, oxygen, amino acids, etc.), produce byproducts (CO2, lactate, formate, etc.), and grow. **E** At the same time, proprietary pharmacokinetics and pharmacodynamics models of the drugs administered account for the dynamics of drug plasma concentrations, uptake by the tissues and tumor, any intracellular conversions that may take place, and overall cytotoxicity. **F**–**G** Because cells in different regions may be growing and/or dying at different rates, the tissues themselves must deform. This is handled through the use of an elastic material mechanics model

This study evaluated a commercially developed multiscale biophysical model that simulates tumor response to various neoadjuvant chemotherapy regimens in three-dimensional space over time [16]. A three-dimensional model of tumorous, vascular, fibroglandular, and fatty tissues is segmented from pre-treatment DCE-MRI for each patient using a UNet-based convolutional neural network, with spatial representations of tumors modeled with cubic voxels of side length 0.5 mm, resulting in approximately 250 K to 550 M voxels per tumor [18, 19]. Initial tumor volume from automated segmentation in this retrospective cohort was reviewed for accuracy by a breast radiologist (H.A.). The simulation is composed of voxel-based cubic lattices of tumor and normal tissue along with chemical concentrations of tumor nutrients (i.e., glucose, oxygen, select amino acids), metabolic byproducts (lactic acid), and chemotherapeutic agents (Fig. 1, Supplemental Figure S1, Supplemental Table S1, Supplemental Methods) [20, 21]. Drug/chemical concentrations are updated over time using an explicit reaction–diffusion equation with sources/sinks defined by a modified Tofts model [20] that captures chemical delivery via microvasculature, and with a macrovascular model incorporating the larger vessels identified on initial segmentation. Tumor growth at each voxel is modeled based on nutrient exposure, and tumor death is modeled based on drug uptake and inhibitory concentrations derived from prior studies (Supplemental Table S2) [22–27]. A mass-spring mesh is used to model the growth/contraction of tumor morphology across voxels. Response is then forecast continuously over small time increments throughout the planned neoadjuvant treatment regimen. Specific inputs are patient age, race, T and N stage, ER percent staining, PR percent staining, HER2 status, grade, histology type, pre-treatment T1-weighted DCE-MRI, and regimen administered. The primary analyses described above were performed with the documented treatment regimen including dose reductions, but model accuracy with standard dose therapy was also evaluated. A predefined cutoff of residual tumor volume less than 0.01 cm^3^ or a 99.9% or greater reduction in tumor volume in these three-dimensional tumor models at the date of surgery was used to predict pCR.

### Statistical analysis

All statistical analyses were performed on individual tumors, with separate predictions performed for each tumor in women with bilateral breast cancers. Baseline clinicopathologic data were compared between the pCR and residual disease subgroups using a chi-squared test for categorical variables and a two-sided two sample t test for continuous variables. The primary outcome metrics of sensitivity and specificity for pCR in the overall population were estimated using the Clopper–Pearson interval [28]. The accuracy of both predicted residual tumor volume and predicted percentage reduction in tumor volume as predictors of pCR was measured with the area under the receiver operating characteristic curve (ROC). As a comparator, a clinical model to predict response was also developed using a logistic regression from patient age, race, disease histology, receptor subtype, tumor grade, tumor T and N stage, and treatment regimen. Logistic regression models were developed on two thirds of the dataset to predict response in the remaining third of the data; the average ROC curve across the entire dataset was calculated using threefold cross validation. Confidence intervals for the area under the ROC curves were computed with 1000 × bootstrapping. To explore the ability of the model to predicted pCR to discriminate long-term outcomes, we compared disease-free interval (DFI), event-free survival (EFS), and overall survival (OS) between predicted pCR and predicted residual disease patients using a log-rank test. The predictions from the biophysical simulation were also correlated with radiographic response, assessed with the Pearson correlation coefficient with significance tested using the Fisher transformation [29]. All statistical testing was done with α = 0.05 significance level. Given the small number of outcomes analyzed and exploratory nature of this analysis, false discovery correction was not performed for these outcomes.

## Results

A total of 233 cases were assessed for eligibility, of which 144 cancers in 141 patients were included for analysis (Supplemental Figure S2). Median patient age was 52 years; the cohort was diverse, with one-half of patients self-reporting Black race. Most cancers were high-grade (77%) invasive ductal carcinomas (96%); 41% of tumors were triple negative. Of patients with HER2-negative breast cancer, 93% received an anthracycline, reflecting the high-risk nature of the population studied. A total of 54% of patients (*n* = 78) received paclitaxel and dose-dense doxorubicin and cyclophosphamide; 22% (*n* = 32) received paclitaxel or docetaxel, carboplatin, trastuzumab with or without pertuzumab (Supplemental Table S3). Median follow-up was 5.4 years; at last follow-up, 11% of patients had recurrence and 14% of patients had died – 9% of whom died from disease. Characteristics did not differ significantly between patients with pCR (*n* = 50) and residual disease (*n* = 94), aside from breast cancer subtype (*p* < 0.001) and recurrence (*p* = 0.005, Table 1).

**Table 1:** Baseline demographics

	Missing	Overall (*n* = 144)	Residual (*n* = 94)	pCR (*n* = 50)	*p*-Value
Age at Diagnosis, mean (SD)	0	51.7 (12.7)	51.4 (12.5)	52.3 (13.3)	0.71
Race, n (%)					
African American	6	72 (50.0)	50 (53.2)	22 (44.0)	0.22
Asian		4 (2.8)	4 (4.3)		
Caucasian		51 (35.4)	28 (29.8)	23 (46.0)	
Native American		1 (0.7)	1 (1.1)		
Other / Missing		16 (11.1)	11 (11.7)	5(10.0)	
Histology, n (%)					
Adenocarcinoma	0	1 (0.7)	0 (0.0)	1 (2.0)	0.21
Invasive Ductal Carcinoma		138 (95.8)	89 (94.7)	49 (98.0)	
Invasive Lobular Carcinoma		4 (2.8)	4 (4.3)	0 (0.0)	
Metaplastic Carcinoma		1 (0.7)	1 (1.1)	0 (0.0)	
Receptor Status n (%)					
HR + /HER2 +	0	24 (16.7)	19 (20.2)	5 (10.0)	< 0.001
HR + /HER2-		36 (25.0)	31 (33.0)	5 (10.0)	
HR−/HER2 +		25 (17.4)	7 (7.4)	18 (36.0)	
TNBC		59 (41.0)	37 (39.4)	22 (44.0)	
Grade, n (%)					
1	0	2 (1.4)	2 (2.1)		0.14
2		31 (21.5)	24 (25.5)	7 (14.0)	
3		111 (77.1)	68 (72.3)	43 (86.0)	
Tumor Stage, n (%)					
T1	0	27 (18.8)	17 (18.1)	10 (20.0)	0.24
T2		86 (59.7)	52 (55.3)	34 (68.0)	
T3		27 (18.8)	22 (23.4)	5 (10.0)	
T4		4 (2.8)	3 (3.2)	1 (2.0)	
Nodal Stage, n (%)					
N0	0	72 (50.0)	43 (45.7)	29 (58.0)	0.57
N1		59 (41.0)	42 (44.7)	17 (34.0)	
N2		10 (6.9)	7 (7.4)	3 (6.0)	
N3		3 (2.1)	2 (2.1)	1 (2.0)	
Regimen, n (%)					
HER2-directed, Anthracycline containing	0	19 (13.2)	11 (11.7)	8 (16.0)	0.41
HER2-directed, Anthracycline free		35 (24.3)	20 (21.3)	15 (30.0)	
Chemotherapy, Anthracycline free		6 (4.2)	5 (5.3)	1 (2.0)	
Chemotherapy, Anthracycline containing		84 (58.3)	58 (61.7)	26 (52.0)	
Months Follow-up, mean (SD)	0	5.6 (2.7)	5.3 (2.7)	5.9 (2.7)	0.21
Alive, n (%)					
Yes	0	123 (85.4)	76 (80.9)	47 (94.0)	0.06
No		21 (14.6)	18 (19.1)	3 (6.0)	
Recurrence, n (%)					
Yes	0	16 (11.1)	16 (17.0)		0.005
No		128 (88.9)	78 (83.0)	50 (100.0)	

*p* values for comparison between cases with pCR and residual disease

Statistics are listed on a per-tumor level, with three cases of bilateral breast cancer

*pCR* Pathologic complete response (ypT0N0 or ypTisN0), *HR* Hormone receptor, *HER2* Human epidermal growth factor receptor 2, *TNBC* Triple-negative breast cancer

**Fig. 2:**
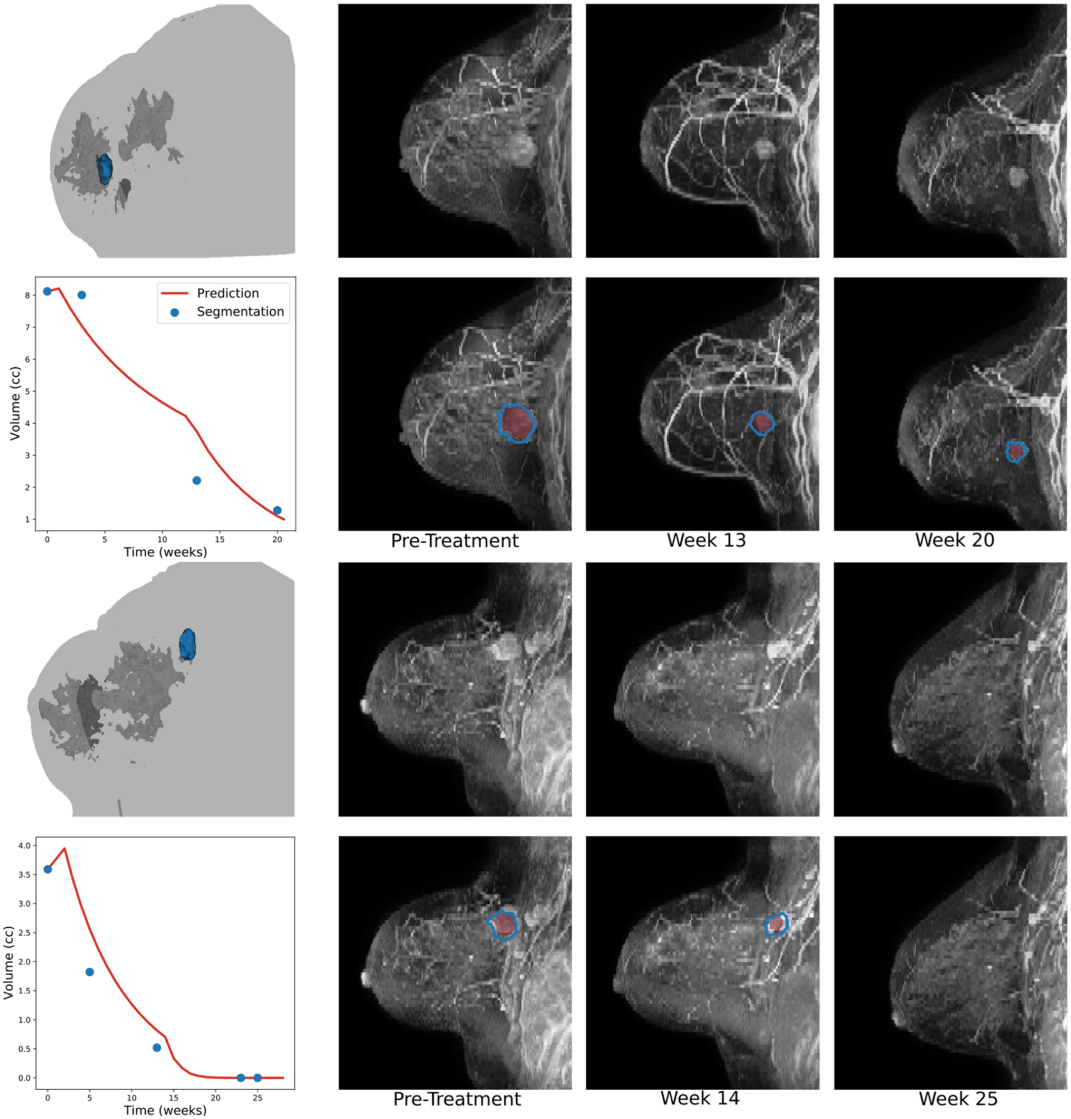
A Biophysical Simulation Predicts Change in Volume and Morphology Throughout Treatment. Representative examples of model predictions showing how the tumor volume changes in response to therapy. A three-dimensional rendering of the breast segmentation derived from DCE-MRI depicting the tumor (blue), glandular tissue (dark gray), and the outline of the patient's body (light gray) at time of diagnosis is shown for each patient. The three-dimensional model is used as input to the model, which predicts change in volume over the treatment course. Predicted tumor volume (red) matches well with segmentations of from intra-treatment MRIs (blue). Examples of patients with residual globular (top) and multi-centric (bottom) tumors are shown, along with an example of a patient achieving pCR (middle)

The biophysical simulation model predicted pCR in 54 patients and residual disease in 90 patients (Table 2), with a sensitivity of 88.0% (95% confidence interval [CI] 75.7 – 95.5), and specificity of 89.4% (95% CI 81.3 – 94.8). Representative volumetric presentations with the model are illustrated in Fig. 2, with simulation results for all patients illustrated in Supplemental Fig. 3. Sensitivity and specificity were preserved across receptor subtypes, with the lowest sensitivity of 80.0% (95% CI 28.4 – 99.5) seen in the HR + /HER2- patients, and the lowest specificity of 86.5% (95% CI 71.2 – 95.5) seen in patients with TNBC. Of the 10 patients predicted to have pCR who had residual disease, 5 had residual nodal disease, and 6 had residual breast disease (Supplemental Table S4). Of the 6 with residual breast tumors, cellularity was available for 4 patients and was ≤ 5% for 3 of the 4 patients. Dose reductions were documented in 28 cases – when predictions were made with standard dose therapy without dose reductions, sensitivity for pCR remained the same, but specificity was reduced to 86.2% (95% CI 77.5—92.4, Supplemental Table S5).

**Table 2:** Outcome Metrics, Overall, and Select Subgroups for Prediction of Pathologic Complete Response

	*n*	n pCR	Accuracy (95% CI)	Sensitivity (95% CI)	Specificity (95% CI)
Overall	144	54	88.9 (82.6–93.5)	88.0 (75.7–95.5)	89.4 (81.3–94.8)
HR + /HER2-	36	7	88.9 (73.9–96.9)	80.0 (28.4–99.5)	90.3 (74.2–98.0)
HER2 +	49	23	91.8 (80.4–97.7)	91.3 (72.0–98.9)	92.3 (74.9–99.1)
TNBC	59	24	86.4 (75.0–94.0)	86.4 (65.1–97.1)	86.5 (71.2–95.5)
Anthracycline-containing regimens	103	40	86.4 (78.2–92.4)	88.2 (72.5–96.7)	85.5 (75.0–92.8)
Anthracycline-free regimens	41	14	95.1 (83.5–99.4)	87.5 (61.7–98.4)	100.0 (86.3–100)

**Fig. 3:**
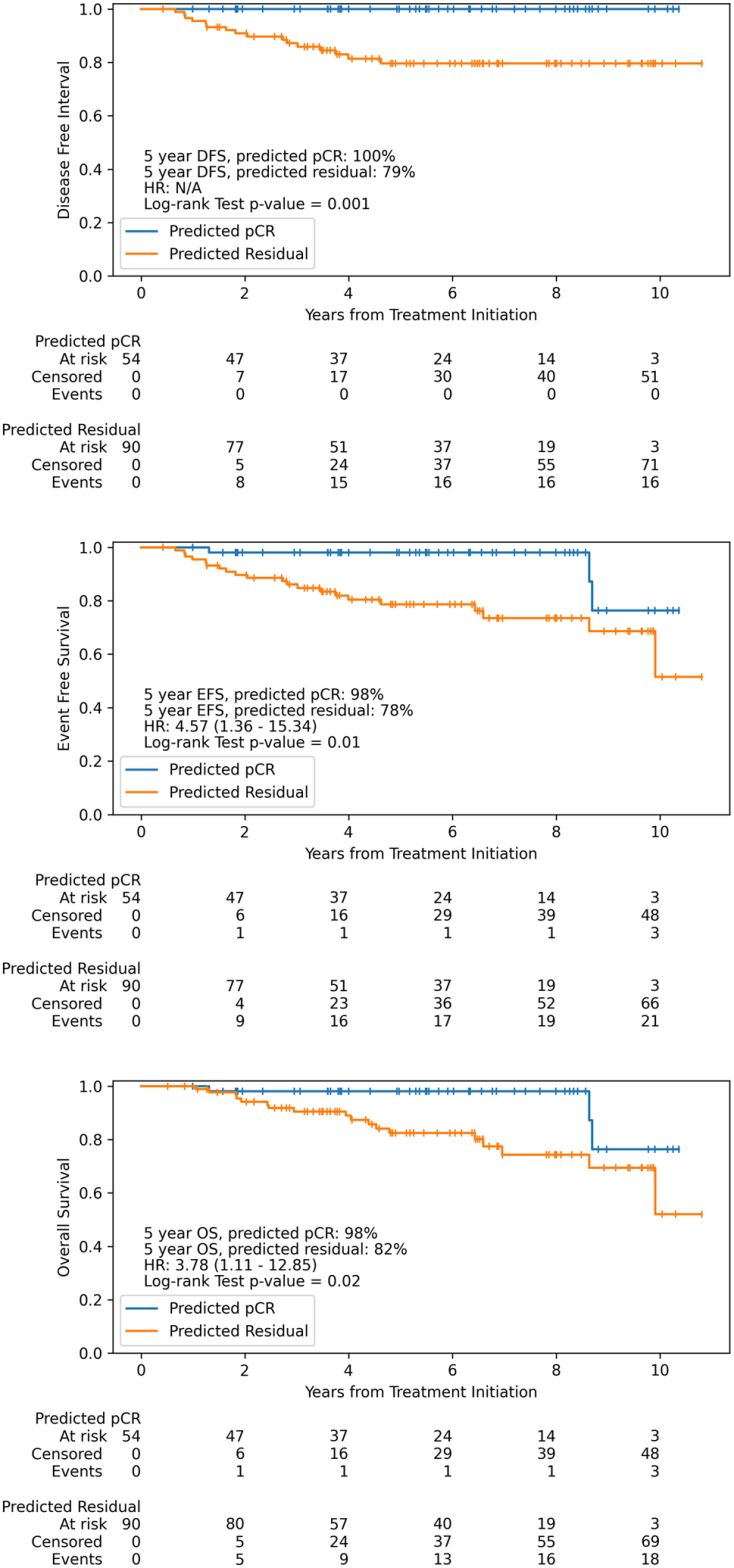
Disease-Free Interval, Event-Free Survival, and Overall Survival in Patients with Predicted Completed Response versus Residual Disease. Patients with predicted complete response as assessed by the biophysical simulation model have improved long-term outcomes compared to patients with predicted residual disease

As pCR predictions are based on a numeric cutoff of simulated residual tumor volume and percent change in tumor volume, we evaluated the discriminative accuracy of these metrics using receiver operating characteristic analysis (Supplemental Figure S4). As a comparator, we used a logistic regression model to predict pCR from all the pre-treatment clinical characteristics used in the biophysical simulation. Simulated percent response and simulated post-treatment volume performed similarly as predictors of response and outperformed a model based on clinical characteristics alone. The AUROC for predicted percent response and predicted post-treatment volume were 0.87 (95% CI 0.80—0.93) and 0.86 (95% CI 0.79—0.93), respectively, compared to 0.72 (95% CI 0.62—0.80) for the logistic regression model using clinical characteristics.

In addition to assessing the ability of the model to accurately predict pCR, we also assessed the correlation of predictions with long-term outcomes. Notably, no patients with predicted pCR experienced disease recurrence (Fig. 3). Patients with predicted pCR had a prolonged DFI (5-year DFI 100% with predicted pCR vs. 79% with predicted residual disease, log-rank *p* = 0.001, HR not computed due to lack of events), prolonged EFS (5-year EFS 98% with predicted pCR vs. 78% with predicted residual disease, log-rank *p* = 0.01, HR 4.57, 95% CI 1.36 – 15.34), and prolonged OS (5-year OS 98% with predicted pCR vs. 82% with predicted residual disease, log-rank *p* = 0.02, HR 3.78, 95% CI 1.11 – 12.85). Biophysical simulation model predictions performed similarly to pCR as a prognostic biomarker (Supplemental Figure S5). Notably, there was a trend towards improved long-term outcomes in patients with residual disease who were predicted to have pCR versus those predicted to have residual disease (Supplemental Figure S6).

**Fig. 4:**
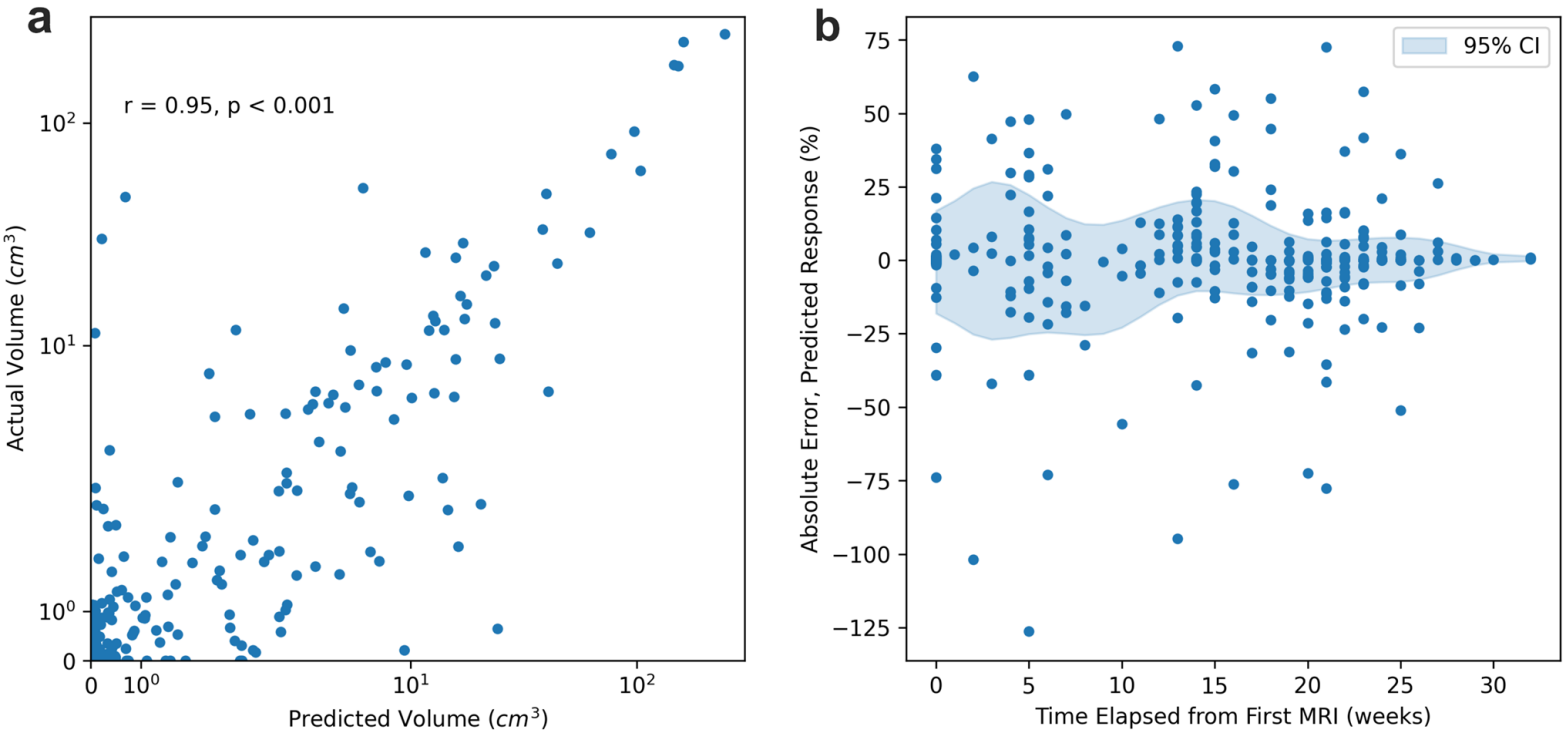
Correlation of Model Predictions and Inter-regimen MRI Volumes. **a** Actual versus predicted tumor volumes, log scale for all inter-regimen scans (*n* = 267 measurements). **b** Absolute error in response over time (*n* = 411, including initial measurements with tumor segmentation), with average and estimated 95% confidence interval illustrated (means and confidence interval with Gaussian filter applied for smoothing)

Aside from predicting pathologic response, model predictions strongly correlated with radiographic assessment of tumor volume for 267 follow-up MRIs, including 144 inter-regimen and 123 post-treatment/pre-operative MRIs (*r* = 0.95, *p* < 0.001, Fig. 4). The mean absolute error in predicted response as a percent of pre-treatment tumor volume for all follow-up MRIs (i.e., excluding initial MRIs that are incorporated into the model) was 9.62% (95% CI 7.94%—11.31%), and the error as a function of time elapsed from initial MRI is illustrated in Fig. 4b. As expected, error in pre-treatment prediction volumes was low – as the pre-treatment MRI was used as model input, and thus, the predicted volume is solely representative of the accuracy of the MRI segmentation algorithm from the model. Nonetheless, absolute error remained < 20% for both inter-regimen and post-treatment scans, which are not used as model inputs (Supplemental Table S6).

## Discussion

In this retrospective, independent validation study, a biophysical simulation model predicted pCR to standard neoadjuvant chemotherapy with a remarkable 89% accuracy. In the small proportion of patients who were predicted to have a pCR but did not, the residual disease was predominantly of low cellularity, providing further evidence that the simulation can accurately model biologic response to chemotherapy. The model’s prediction of response distinguishes patients with good and poor long-term prognosis in this retrospective cohort, with no recurrences in patients with predicted pCR. Although the ability to test the model with different regimens in a retrospective setting is limited by the dosing schedules and combinations that are currently used in clinical practice, comparison of predicted volume to inter-regimen MRI suggest that the tool is accurately modeling response to individual drugs. Predictive accuracy was similar across tumor subtypes and treatment regimens. Additionally, this retrospective study demonstrated admirable performance of a predictive model in an ethnically/racially diverse cohort, enriched for African–American patients, who are uniquely affected by aggressive triple-negative breast cancers but often underrepresented in clinical trials [30].

Other multiscale models of breast cancer response to therapy have been evaluated in silico [16, 31] in small exploratory cohorts of patients [32]. To our knowledge this is the first large-scale independent validation of a multiscale simulation model of response to breast cancer treatment. Other models using pre-treatment imaging data have generally relied on extraction of radiomic features, and most have not been externally validated [33]. In one radiomic model with external validation in three institutions, AUROCs ranged from 0.71 to 0.80 [9]. Deep learning, a form of artificial intelligence, has also been used to predict response to neoadjuvant therapy from MRI – in one study, response prediction for HER2-directed therapy achieved AUROCs of 0.77 and 0.85 in two validation cohorts [34]. The biophysical simulation model compares favorably to these other tools, with an overall accuracy, sensitivity, and specificity of approximately 90%. Since the prediction of pCR from the simulation is made based on a threshold of both percent response and residual tumor volume, it is not possible to construct a precise AUROC curve to directly compare to other models. However, AUROC for percent response (0.87) and residual tumor volume (0.86) in this external cohort compare favorably to other radiology-based models of response (Fig. 3). The biophysical simulation model also predicted response more accurately than clinical factors alone. This is consistent with other recently published work, demonstrating an AUROC of 0.70 for prediction of pCR using clinical risk factors [35]. Although hormone receptor and HER2 status remain the most important features for response prediction, the simulation of response with a biophysical model adds incremental accuracy, and further study to identify model parameters most predictive of response is ongoing.

Anthracycline use in early breast cancer carries a dose-dependent risk of both long-term cardiac toxicity [36] and secondary hematologic malignancies [37]. As this biophysical simulation model can precisely predict response to specific NAC regimens, it could profoundly impact clinical care, by identifying patients who can achieve a pCR with an anthracycline-sparing regimen, thereby minimizing the risk of long-term toxicities in individuals who do not need such cardiotoxic therapies. And, while immunotherapy has demonstrated efficacy in early TNBC, the additive pCR benefit ranges from 8% in KEYNOTE-522 [38] to 17% in IMpassion031 [7]. Thus, the majority of patients do not need or will not benefit from immunotherapy, exposing a large number of patients to the risk of long-term immune-related toxicities for not benefit. Unlike in metastatic TNBC, PD-L1 is not a predictive biomarker of response in the neoadjuvant setting; a biophysical simulation model can accurately identify patients who would have a pCR with standard neoadjuvant chemotherapy, thus, sparing a significant proportion exposure to immunotherapy. Such a model could also be used to further delineate the benefit of emerging therapies tested in the neoadjuvant setting.

Our analysis of this biophysical simulation model had several limitations. In patients who received treatment locally, dose reductions and treatment delays were incorporated into the simulation. This analysis reflects a feasible use of the model in clinical practice – for example, if a dose reduction is considered, the tumor response could be re-analyzed with the modified regimen to determine if the dose adjustment resulted in conversion from predicted pCR to predicted residual disease. Given the retrospective nature of this study, it is not possible to determine the exact pre-treatment regimen planned for all patients with complete confidence. Nonetheless, we also analyzed model performance without dose reductions, demonstrating a minimal reduction in model specificity, and highlighting that model performance is maintained using purely pre-treatment data.

The biophysical model evaluated in this study predicts response with a simulation of the primary breast tumor but does not separately model response in lymph nodes. Four patients with predicted pCR had ypT0/Tis response with residual lymph node disease at the time of surgery. Models that simulate response in lymph nodes along with the primary tumor bed may allow for even more accurate stratification of pCR, especially as some studies suggest radiomic features of lymph nodes are most predictive of response [11].

## Conclusions

A biophysical simulation model predicted pCR in this independent single institution validation cohort with remarkable accuracy. False-positive predictions of response generally occurred in cases of minimal residual cellularity, and no recurrences were seen in patients with a predicted pathologic complete response. This model could be used to identify patients eligible for both de-escalation and escalation of treatment, allowing clinicians to treat patients with immunotherapy or other novel agents if they are predicted to respond poorly to standard NAC. With better prediction of pathologic response and long-term outcomes, multiscale simulation models can usher in an era of personalized medicine for patients with high-risk, early-stage breast cancer.

## Supplementary Information

Below is the link to the electronic supplementary material.

Supplementary file1 (CSV 48 kb)

Supplementary file2 (DOCX 3287 kb)

## Data Availability

Anonymized clinical data and results of response prediction are available as a supplement to this manuscript.
