# Supplementary material for: Highly accurate response prediction in high-risk early breast cancer patients using a biophysical simulation platform
Source: Breast Cancer Res Treat. 2022 Sep 5;196(1):57–66. doi: 10.1007/s10549-022-06722-0 (PMC9550684; doi:10.1007/s10549-022-06722-0)
Supplement: Supplementary file 2 — Supplementary file2 (DOCX 3287 kb) [file 10549_2022_6722_MOESM2_ESM.docx]

**Supplemental Information**

**Highly Accurate Response Prediction in High-Risk Early Breast Cancer Patients Using a Biophysical Simulation Platform**

Frederick M. Howard^1^, Gong He^1^, Joseph R. Peterson^2^, JR Pfeiffer^2^, Tyler Earnest^2^, Alexander T. Pearson^1^, Hiroyuki Abe^3^, John A. Cole^2*^, Rita Nanda^1*^

^1^ Department of Medicine, University of Chicago, Chicago, IL, USA

^2^ SimBioSys, Chicago, IL, USA

^3^ Department of Radiology, University of Chicago, Chicago, IL, USA

*These authors contributed equally to the manuscript

# **Supplemental Methods**

# **1. Data Extraction Procedure and Simulation Inputs**

The biophysical simulation model takes as input a variety of standard-of-care demographic, pathological, and radiological information such as age, race, ER/PR/HER2 status, ER/PR staining percentage, HER2 IHC Score or ISH measurement, cancer grade, cancer subtype and cancer stage, BRCA1 mutational status, and fat suppressed T1-weighted dynamic contrast enhanced (DCE) magnetic resonance images (MRI). In addition, the prescribed neoadjuvant therapy is input. Clinical notes, pathology reports, and medical imaging for patients eligible for the study were extracted from the EMR/PACS system and de-identified by F.H., G.H., and H.A. J.R.Pf. extracted relevant information from the clinical notes and pathology reports. MRIs were processed automatically via the biophysical simulation model.

# **2. Model Methods**

# **2.1 Segmentation of MRI**

DCE MRI data is input into a segmentation module of the model to produce a “segmentation” of the tumor and surrounding tissues. The segmentation is a three-dimensional cubic lattice of integer “labels” that describe the dominant tissue within the voxel. Seven labels are included in the segmentation: air, skin, fat, gland, vasculature, tumor, and torso.

Image segmentation is performed by a convolutional neural network (CNN). The network architecture is 3D Res-UNet^1–3^ that segments DCE MRI at a 1 mm^3^ resolution pre-trained using radiologist segmented images from public datasets external. Multiple frames (timepoints) from the DCE MRI, including one pre-contrast and multiple post-contrast, are interpolated to a 1 mm^3^ prior to input into the CNN for prediction of the label lattice.

The resulting label lattice is then upsampled to 0.5 mm^3^ and input into the simulation as the initial shape and organization of the tumor and healthy tissue. An example segmentation can be seen in Supplemental Figure 1.


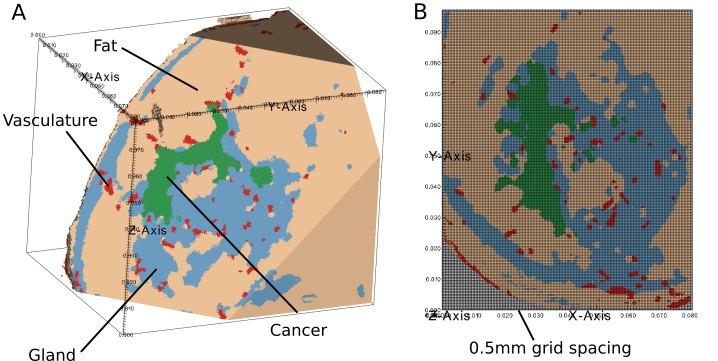


**Supplemental Figure 1. A)** Representative 3D visualization of a CNN-segmented label map with a cutaway through the breast to highlight the cancerous region. Four labelled regions used in the simulation are shown including fatty, glandular, vascular, and cancerous tissues. **B)** Representative cross-section through the segmentation to demonstrate the 0.5 mm^3^ spatial discretization used in the simulation.

# **2.2 Simulation Algorithm**

The simulation comprises cubic lattices of chemical concentrations (*C*) and tissue volume fractions (*θ*). Chemicals include nutrients used by the tissues, metabolic byproducts, and drugs. Tissue volume fractions represent the amount of tissue at each location as the cancer grows and dies. Chemical concentrations evolve according to the reaction-diffusion partial differential equation (PDE):

$$\partial_{t}C = D \nabla^{2}C + R_{C}\left( \overline{C}, \theta\right)$$

where *D* is the diffusion coefficient of the chemical, and $R\left( \overline{C}, \theta\right)$, the “reaction term”, is a term describing the production and consumption of chemical C via biological processes at each location. For each chemical species, C, $R_{C}\left( \overline{C}, \theta\right)$ represents the temporal rate of change of C due to all reactions in the simulation, including metabolic reactions. It is assumed that only tissues perform reactions, and thus, $R_{C}\left( \overline{C}, \theta\right)$ will be proportional to $\theta$. Several different non-metabolic reactions can be performed, including first-order, second-order, and Michaelis-Menten type reactions. These are usually associated with drug uptake/efflux or other pharmacodynamic events in our simulations. For concreteness, we can consider a drug that is not metabolized. Let’s assume this drug, C, is taken up by the local tissue through a Michaelis-Menten-type reaction, transforming it to an intracellular form, C_i_, and that this intracellular form can either be effluxed again through another Michaelis-Menten type reaction, or further react through a first-order reaction to form some active form, C_a_, which might be degraded by the cells. In this case the exact forms of $R_{C}\left( \overline{C}, \theta\right)$, $R_{C_{i}}\left( \overline{C}, \theta\right)$, and $R_{C_{a}}\left( \overline{C}, \theta\right)$ would be:

$$R_{C}\left( \overline{C}, \theta\right)= \frac{-\theta K_{max}^{uptake} C}{K_{M}^{uptake} + C} + \frac{\theta K_{max}^{efflux} C_{i}}{K_{M}^{efflux} + C_{i}}$$

$$R_{C_{i}}\left( \overline{C}, \theta\right)= \frac{\theta K_{max}^{uptake} C}{K_{M}^{uptake} + C} - \frac{\theta K_{max}^{efflux} C_{i}}{K_{M}^{efflux} + C_{i}} - \theta k^{activate} C_{i}$$

$$R_{C_{a}}\left( \overline{C}, \theta\right)= \theta k^{activate} C_{i} - \theta k^{\deg rade} C_{a}$$

Metabolic reactions are handled somewhat differently; these utilize the result of flux balance analysis, which is described in 2.3 below.

Diffusion is modeled via the finite difference method^4^. The reaction term includes contributions from cancer metabolism, drug pharmacokinetics (PK), and drug pharmacodynamics (PD) as described in subsequent sections. The PDE is numerically solved via the explicit forward Euler method^5^ with a timestep selected to ensure numerical convergence.

The tissue is represented via a mass and spring mesh (Hooke’s law):


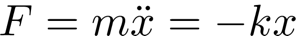
,

where *m* is the mass, *k* is a tissue-specific spring constant, and *x* is the location of the mass. At specified intervals throughout the simulation, the forces are computed, and the tissue allowed to relax iteratively. The relaxed spring mesh is re-interpolated to the same lattice as the chemical concentrations to update the volume fractions, *θ*. The volume fraction grows or shrinks in response to cancer metabolism as described below.

# **2.3 Cancer Metabolism Modeling**

Cancer metabolism is modeled via a modeling approach known as flux balance analysis (FBA)^6^ . Briefly, let S be the stoichiometric matrix representing all metabolic biochemical reactions carried out by a cell, such that S_i,j_ is the stoichiometry of metabolite i involved in reaction j. For example, if reaction j cleaves one molecule of metabolite k into two molecules of metabolite l, then S_k,j_ = -1 and S_l,j_ = 2. Further, let there exist some “biomass" reaction in S that transforms building blocks like amino acids, nucleic acids, lipids, etc. into a unit of new cellular material. Let v represent the vector of fluxes through each reaction (in suitable units). Then the flux through the biomass reaction represents the proliferation rate of the cells. Assuming that within a cell, the concentrations of all metabolites are at steady-state (meaning they do not build up and disappear indefinitely), then $Sv =\frac{d\overline{C}}{dt}= 0$. Naturally, some of the reactions in S represent the uptake of extracellular metabolites like glucose or oxygen. Constraints can be set on the fluxes through these reactions such that the modeled cells cannot take up more than is available within their local voxel. In our case, such a bound can be set based on the concentration of the metabolite in a voxel and the timestep of the algorithm, e.g. $v_{uptake} \leq\frac{C}{\Delta t}$. Other bounds may also exist that represent the gene expression state of the cells. For example, if reaction j is catalyzed by some enzyme E, and E is not expressed by the cells, then v_j_ = 0. The question then becomes, what is the optimal set of fluxes given all constraints? If we assume cancer cells are primarily programmed to proliferate, then we can use the technology of linear programming to solve the problem of maximizing v_bm_ subject to the constraints $Sv = 0, lb \leq v \leq ub$, where v_bm_ is the flux through the biomass equation, lb is the vector of flux lower bounds, and ub is the vector of flux upper bounds. Of course, in the case of non-cancerous tissues, proliferation may not be the most reasonable objective. But we still expect some metabolic activity in order to sustain life. In such cases it is reasonable to assume the cells require some maintenance ATP generation (imposed through a reaction that consumes ATP with some positive flux lower bound), and that their optimal metabolic behavior is the one that minimized total flux through the reaction network (parsimonious FBA, in the parlance of the field).

Given the concentrations of metabolites in a voxel, and the local densities of the tissue, flux vectors for each tissue can be found. From there, both the proliferation rate, $\gamma$, and the uptake/efflux rates of each metabolite by each tissue can be simply read off as $\gamma= v_{bm}$ and $\frac{dC}{dt}=M_{c} = \theta v_{C}$, where v_C_ is the flux through the uptake/efflux reaction for metabolite C. Importantly, these later rates form the "metabolic" part of $R\left( \overline{C}, \theta\right)$ described above. Had the drug we considered previously in section 2.2 also been metabolized by the local cells, $R\left( \overline{C}, \theta\right)$ might have looked like:

$$R_{C}\left( \overline{C}, \theta\right)= \frac{-\theta K_{max}^{uptake} C}{K_{M}^{uptake} + C} + \frac{\theta K_{max}^{efflux} C_{i}}{K_{M}^{efflux} + C_{i}} +M_{C}$$

For each major histological subtype (HR+/HER2-, HR+/HER2+, HR-/HER2+, and TNBC) a different set of constraints are used to reflect differences in the gene expression states of the subtypes. These are based on transcriptomics profiles taken from the TCGA^7^. The gimm3e algorithm^8^ was used to set appropriate bounds on the internal fluxes for each subtype. In addition, for each subtype, different nutrients are tracked (see Supplemental Table 1). The choice of tracked metabolites was based on a sensitivity analysis of growth of the cancerous tissue.

**Supplemental Table 1. Metabolites Tracked within the Simulation**

| **Histological Subtype** | **Tracked Metabolites** |
| --- | --- |
| **HR+/HER2-** | Glucose, Oxygen, Glycine, Glutamine, Lactic acid, Glycine, Uracil, Uridine, Glutathione |
| **HR+/HER2+** | Glucose, Oxygen, Lactic acid, Proline, α-Ketoglutaric acid, Guanine |
| **HR-/HER2+** | Glucose, Oxygen, Lactic acid, Alanine, Uridine, Uridine monophosphate |
| **TNBC** | Glucose, Oxygen, Lactate, Alanine, Uridine, Uridine monophosphate |

# **2.4 Drug Modeling**

Drugs are a special type of chemical within the simulation. Three types of proprietary models account for drugs and their effects relating to PK, PD, and mechanism of action (MoA) in the simulation. The PK models represent the systemic plasma concentrations and distribution to the simulated tissues. Within our simulations, the plasma concentrations are assumed to follow pre-specified time courses. In most cases these time courses have single- or tri-exponential forms and are taken directly from the literature^9–16^. As such, they represent an average patient. A regimen in which a given drug is administered at some dose and on some schedule will thus have an associated "spike train" of plasma concentrations consisting of a rapid rise immediately after each administration followed by a gradual decrease until the next administered dose. In the present study, each drug’s plasma concentration dynamics were specified for each patient based on the regimen they were prescribed by their physician, and explicitly accounted for the doses and their dates (relative to the start of treatment). The distribution of the drugs to the simulated tissues is accomplished via diffusion from the macroscopic vasculature and by way of a proprietary “microvascular model,” described below in Section 2.5. The PD models describe the dynamics of the intracellular (or, in the case of antibodies like trastuzumab or pertuzumab, bound) concentrations of drugs. In most cases these models will involve chemical reactions that describe drug uptake and efflux (or binding and unbinding) as Michaelis-Menten kinetic equations, but may also include additional intracellular conversions, such as the hydration of carboplatin which are first order. Finally, the intracellular (or bound) drugs contribute to reduced proliferation and cell death in a concentration-dependent manner. Equations and parameterizations thereof were fitted to data published in the literature^17–27^. For most drugs within our simulations, including the anthracyclines, taxanes, antibodies, platins, and cyclophosphamide modeled in this study, cell proliferation is described in terms of a viable and non-viable fraction. Viability decreases with increasing drug concentration according to:

$$v = \frac{1}{1 + \frac{D}{IC_{50}}}$$

where *v* represents the viable fraction, D represents the intracellular drug concentration, and IC_50_ represents the concentration at which half the cells become non-viable. The viable fraction is assumed to grow at rate $\gamma$(see SI Section 2.3 above) while the non-viable fraction, 1 – *v,* is assumed to die at some rate.

# **2.5 Vascular Modeling**

Chemicals enter and are cleared from the system via macroscopic and microscopic vascular, *i.e.*, they act as boundary conditions. Macroscopic vasculature is derived from the segmentation model. Microscopic vasculature is modeled via a proprietary model (aka “microvascular model”) that shares characteristics with the Tofts model^28^. A vascular density and rate of leakiness is determined at each position in the tissue by fitting the microvascular model with the DCE MRI data. These parameters are then used to model the appearance and disappearance of each chemical at every location in the simulation according to:

$$\partial_{t}C=KP1 C_{p} - KP2 C$$

where C_p_ represents the plasma concentration of a given chemical, and KP1 and KP2 are parameters derived from the microvascular model that are analogous to K_trans_ and K_ep_ from the standard Tofts model, respectively.

The macroscopic and microscopic vascular models are not coupled within the simulation. Macroscopic vasculature is treated as a volume fraction like the other three tissues and can move around within the simulation space in response to growth and death of the tumor. Functionally, the macroscopic vasculature acts as a constant source and sink for nutrients which are assumed to be invariant over time (constant Dirichlet boundary condition), and as a time varying source and sink for drugs as prescribed by the pharmacokinetic equations (time varying Dirichlet boundary condition).

The effect of the microscopic vasculature at each location is determined by the equation above and updated at each timestep. Thus, the concentration of drugs and nutrients within a voxel are a function of both the effects of nearby macroscopic vasculature and the local microscopic vasculature.

**2.6 Model Parameterization**

Model parameters are either fixed across all breast cancer patients (“universal constants”) or are parameterized for each major histological subtype (HR+/HER2-, HR+, HR-/HER2+, and TNBC). Universal constants include the diffusion coefficients of chemicals and the pharmacokinetics of drugs in the blood stream (see Supplemental Table 2). Diffusion coefficients of drugs and nutrients were taken from literature or estimated from molecular mass^29^. Pharmacokinetic parameters were taken from the literature^9–16^. Parameters found in metabolic models, drug uptake/efflux, and tumor susceptibility to drugs (e.g., IC0) are fitted on a per-histological subtype basis. These were fitted to publicly available patient datasets^30,31^ using standard non-linear fitting methods (as described previously^32–35^) to minimize the residual error between the simulation prediction of tumor volume and tumor volume assessed from DCE-MRIs taken longitudinally throughout treatment (generally 1-2 timepoints during treatment, and 1 timepoint after treatment but prior too surgery).

**Supplemental Table 2. Drug Parameters**

| **Drug** | **PK Model Parameters** | **IC_50_ Parameter** | **Diffusion Coefficient (μm^2^/s)** | **Molecular Weight (g/mol)** |
| --- | --- | --- | --- | --- |
| **Carboplatin** | Tri-Exponential  A=0.40, a=0.17 hr^-1^  B=0.54, b=1.4 hr^-1^  C=0.068, c=22 hr^-1^ | 66 µM | 640 | 373.27 |
| **Cisplatin** | Tri-Exponential  A=0.24, a=0.60 hr^-1^  B=0.19, b=0.60 hr^-1^  C=0.58, c=67.5 hr^-1^ | 40 µM | 640 | 300.01 |
| **Cyclophosphamide** | Single Exponential  a=0.077 hr^-1^ | 57 nM | 100 | 261.07 |
| **Docetaxel** | Single Exponential  a=0.051 hr^-1^ | 7.5 nM | 100 | 807.88 |
| **Doxorubicin** | Single Exponential  a=0.021 hr^-1^ | 7.9 µM | 633 | 543.54 |
| **Paclitaxel** | Single Exponential  a=0.050 hr^-1^ | 46 nM | 100 | 853.91 |
| **Pertuzumab** | Single Exponential  a=0.0016 hr^-1^ | 50 µM | 780 | 148088.0 |
| **Trastuzumab** | Single Exponential  a=0.0011 hr^-1^ | 50 µM | 780 | 145531.5 |

# **3. Image Segmentation Validation**

Tumor regions of interest (ROIs) at each of the clinical timepoints were hand delineated by J.R.Pf using a proprietary tool customized for DCE-MRI. This process was performed only after the simulation results were submitted to the clinical team and “locked” for analysis, but prior to returning the results to the team at SimBioSys. H.A. verified the accuracy of the segmentations. In situations where the tumor ROI was determined to be incorrect, the segmentations were corrected. This process was performed until consensus was met. Only after this process was completed were statistics describing volumetric accuracy of the simulations computed.

**Supplemental References**

1. Ronneberger, O., Fischer, P. & Brox, T. U-Net: Convolutional Networks for Biomedical Image Segmentation. (2015).

2. Zhang, Z., Liu, Q. & Wang, Y. Road Extraction by Deep Residual U-Net. *IEEE Geoscience and Remote Sensing Letters* **15**, 749–753 (2018).

3. Kerfoot, E. *et al.* Left-Ventricle Quantification Using Residual U-Net. in 371–380 (2019). doi:10.1007/978-3-030-12029-0_40.

4. Grossmann, C., Roos, H.-G. & Stynes, M. *Numerical Treatment of Partial Differential Equations*. (Springer Berlin Heidelberg, 2007). doi:10.1007/978-3-540-71584-9.

5. Butcher, J. *Numerical Methods for Ordinary Differential Equations*. (Wiley, 2008).

6. Orth, J. D., Thiele, I. & Palsson, B. Ø. What is flux balance analysis? *Nature Biotechnology* **28**, 245–248 (2010).

7. Tomczak, K., Czerwińska, P. & Wiznerowicz, M. Review The Cancer Genome Atlas (TCGA): an immeasurable source of knowledge. *Współczesna Onkologia* **1A**, 68–77 (2015).

8. Schmidt, B. J. *et al.* GIM3E: condition-specific models of cellular metabolism developed from metabolomics and expression data. *Bioinformatics* **29**, 2900–2908 (2013).

9. Oguri, S. *et al.* Clinical Pharmacokinetics of Carboplatin. *The Journal of Clinical Pharmacology* **28**, 208–215 (1988).

10. Urien, S. & Lokiec, F. Population pharmacokinetics of total and unbound plasma cisplatin in adult patients. *British Journal of Clinical Pharmacology* **57**, 756–763 (2004).

11. Juma, F., Rogers, H. & Trounce, J. Pharmacokinetics of cyclophosphamide and alkylating activity in man after intravenous and oral administration. *British Journal of Clinical Pharmacology* **8**, 209–217 (1979).

12. Kenmotsu, H. & Tanigawara, Y. Pharmacokinetics, dynamics and toxicity of docetaxel: Why the Japanese dose differs from the Western dose. *Cancer Science* **106**, 497–504 (2015).

13. Hoffmann, M., Bergner, R., Stützle, M., Uppenkamp, M. J. & Foerster, R. Pharmacokinetics of Doxorubicin In Normal Weight and Obese Lymphoma Patients. *Blood* **116**, 4935–4935 (2010).

14. Ohtsu, T. *et al.* Clinical pharmacokinetics and pharmacodynamics of paclitaxel: a 3-hour infusion versus a 24-hour infusion. *Clin Cancer Res* **1**, 599–606 (1995).

15. Agus, D. B. *et al.* Phase I Clinical Study of Pertuzumab, a Novel HER Dimerization Inhibitor, in Patients With Advanced Cancer. *Journal of Clinical Oncology* **23**, 2534–2543 (2005).

16. Bruno, R. *et al.* Population pharmacokinetics of trastuzumab in patients With HER2+ metastatic breast cancer. *Cancer Chemotherapy and Pharmacology* **56**, 361–369 (2005).

17. Ghezzi, A., Aceto, M., Cassino, C., Gabano, E. & Osella, D. Uptake of antitumor platinum(II)-complexes by cancer cells, assayed by inductively coupled plasma mass spectrometry (ICP-MS). *Journal of Inorganic Biochemistry* **98**, 73–78 (2004).

18. Goldenberg, G. J., Land, H. B. & Cormack, D. v. Mechanism of cyclophosphamide transport by L5178Y lymphoblasts in vitro. *Cancer Res* **34**, 3274–82 (1974).

19. Kuh, H. J., Jang, S. H., Wientjes, M. G. & Au, J. L. Computational model of intracellular pharmacokinetics of paclitaxel. *J Pharmacol Exp Ther* **293**, 761–70 (2000).

20. Lavelle, F. *et al.* Preclinical evaluation of docetaxel (Taxotere). *Semin Oncol* **22**, 3–16 (1995).

21. El-Kareh, A. W. & Secomb, T. W. Two-Mechanism Peak Concentration Model for Cellular Pharmacodynamics of Doxorubicin. *Neoplasia* **7**, 705–713 (2005).

22. Maadi, H., Nami, B., Tong, J., Li, G. & Wang, Z. The effects of trastuzumab on HER2-mediated cell signaling in CHO cells expressing human HER2. *BMC Cancer* **18**, 238 (2018).

23. Larsen, S. K., Gao, Y. & Basse, P. H. NK Cells in the Tumor Microenvironment. *Critical Reviews in Oncogenesis* **19**, 91–105 (2014).

24. Hoffman, F. *et al.* A mathematical model of antibody-dependent cellular cytotoxicity (ADCC). *Journal of Theoretical Biology* **436**, 39–50 (2018).

25. Bostrom, J., Haber, L., Koenig, P., Kelley, R. F. & Fuh, G. High Affinity Antigen Recognition of the Dual Specific Variants of Herceptin Is Entropy-Driven in Spite of Structural Plasticity. *PLoS ONE* **6**, e17887 (2011).

26. Adams, C. W. *et al.* Humanization of a recombinant monoclonal antibody to produce a therapeutic HER dimerization inhibitor, pertuzumab. *Cancer Immunology, Immunotherapy* **55**, 717–727 (2006).

27. Scheuer, W. *et al.* Strongly Enhanced Antitumor Activity of Trastuzumab and Pertuzumab Combination Treatment on HER2-Positive Human Xenograft Tumor Models. *Cancer Research* **69**, 9330–9336 (2009).

28. Tofts, P. S. & Kermode, A. G. Measurement of the blood-brain barrier permeability and leakage space using dynamic MR imaging. 1. Fundamental concepts. *Magnetic Resonance in Medicine* **17**, 357–367 (1991).

29. Kim, S. *et al.* PubChem 2019 update: improved access to chemical data. *Nucleic Acids Research* **47**, D1102–D1109 (2019).

30. Huang, W. *et al.* Variations of Dynamic Contrast-Enhanced Magnetic Resonance Imaging in Evaluation of Breast Cancer Therapy Response: A Multicenter Data Analysis Challenge. *Translational Oncology* **7**, 153–166 (2014).

31. Newitt, D. & Hylton, N. Single site breast DCE-MRI data and segmentations from patients undergoing neoadjuvant chemotherapy. *The Cancer Imaging Archive* (2016).

32. Cole, J. A., Peterson, J. R., Earnest, T. M., Hallock, M. J. & Braun, E. Abstract P1-06-04: SimBioSys TumorScope: Spatio-temporal modeling of the breast tumor microenvironment accurately predicts chemotherapeutic response. *Cancer Research* **80**, P1-06-04-P1-06–04 (2020).

33. Cole, J. A. *et al.* SimBioSys TumorScope: Spatio-temporal modeling of the tumor microenvironment to predict chemotherapeutic response. *Journal of Clinical Oncology* **38**, e12650–e12650 (2020).

34. Mamillapalli, C. K. *et al.* Spatiotemporal modeling with SimBioSys TumorScope to predict chemotherapeutic response in breast tumor microenvironments. *Journal of Clinical Oncology* **38**, e12656–e12656 (2020).

35. Cole, J. A. *et al.* Abstract P1-08-31: Simbiosys tumorscope: Biophysical modeling of patient-specific response to chemotherapy. *Cancer Research* **82**, P1-08-31-P1-08–31 (2022).

**144** Included Cases

**Supplemental Figure 2. CONSORT diagram illustrating breast tumors included in the study.**


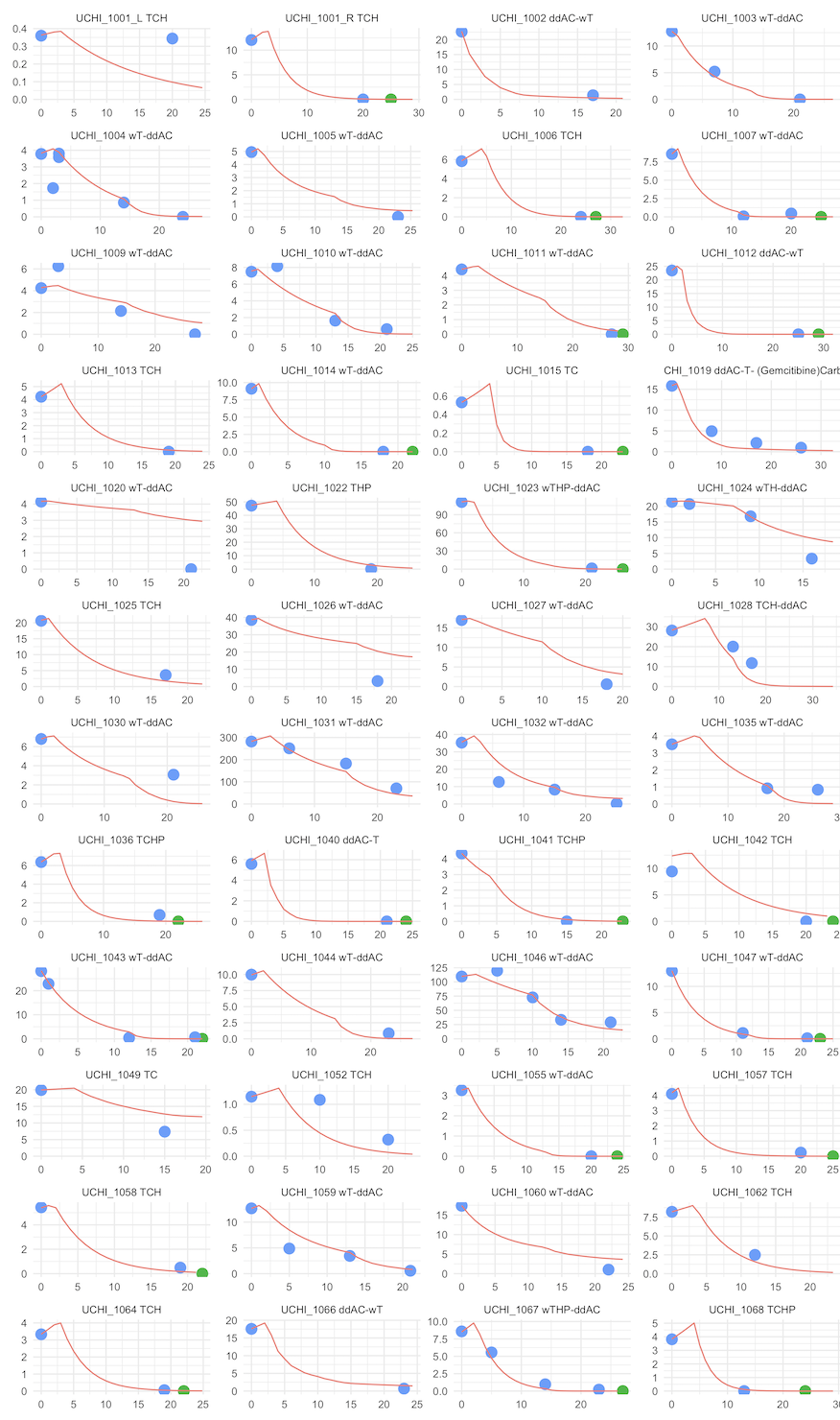


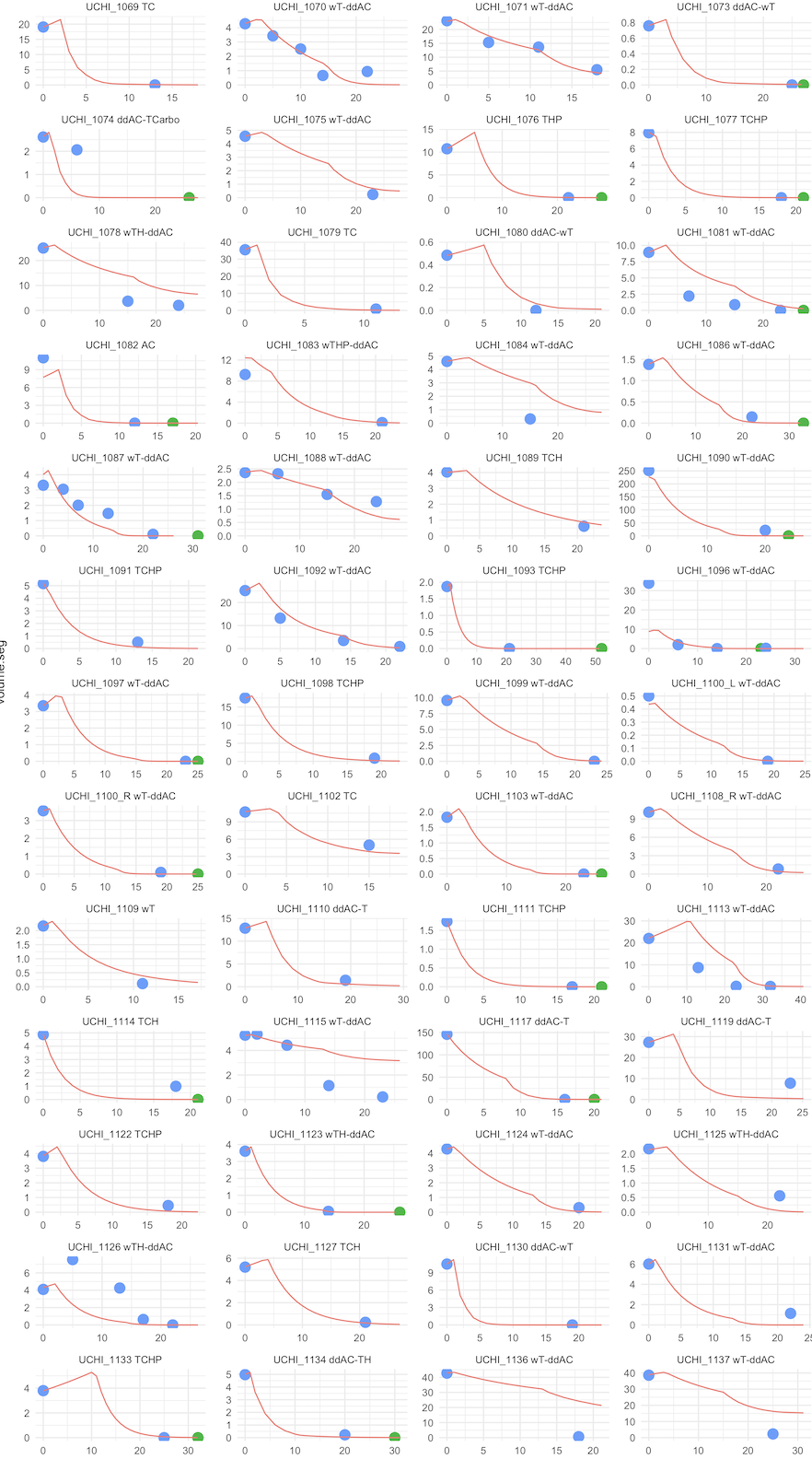


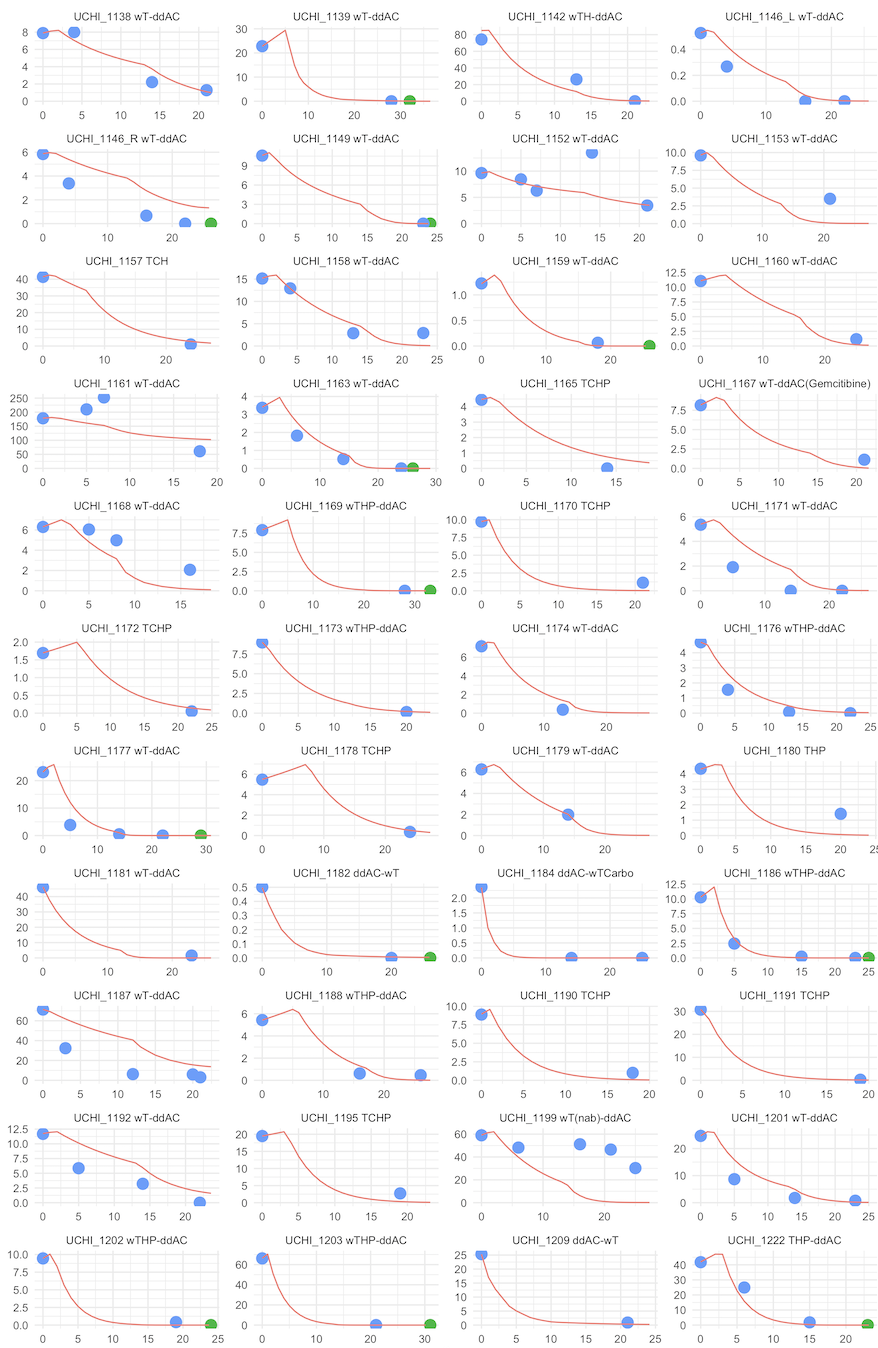


**Supplemental Figure 3. Comparison of Model Predictions with Actual Tumor Volumes.** Predicted Traces of Volume in cubic centimeters (Orange Line) on y-axis over weeks of NACT (x-axis). Presented with ground truth either radiographic (volume from MRIs in blue) and pathologic where patient achieved pCR (green).


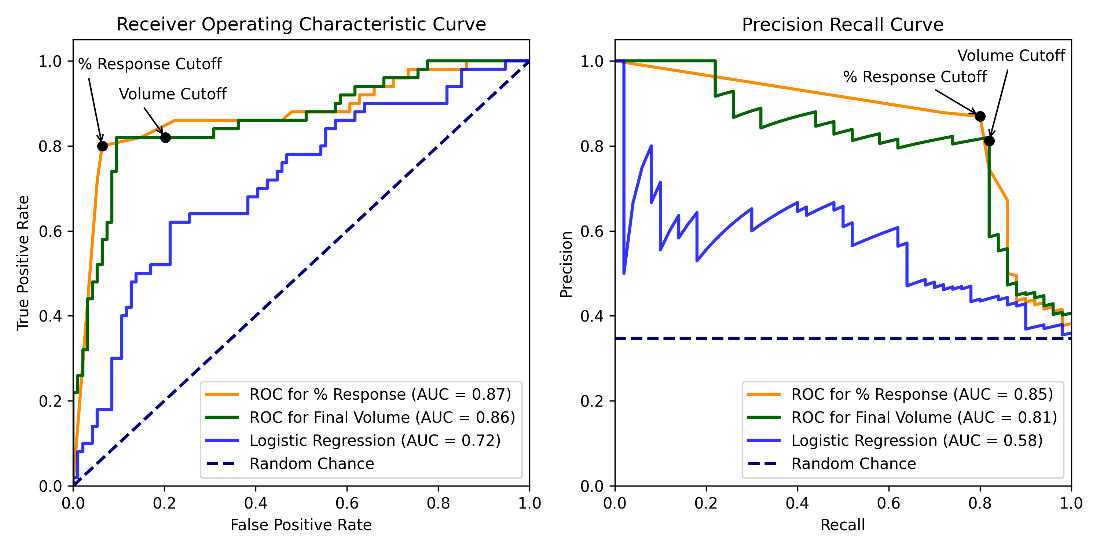


**Supplemental Figure 4. Receiver Operating Characteristic Curve and Precision Recall Curve for Response Prediction.** Curves illustrated for both percent response in tumor volume and by residual tumor volume predicted by the biophysical simulation model in this independent validation cohort. Predefined percent response and volume cutoffs used for determination of pCR are depicted. Also depicted is the average receiver operating characteristic and precision recall curve for a logistic regression model predicting response from age, ethnicity, receptor subtype, grade, tumor / nodal stage, histologic subtype, and regimen received, generated with 3-fold cross validation within this dataset.


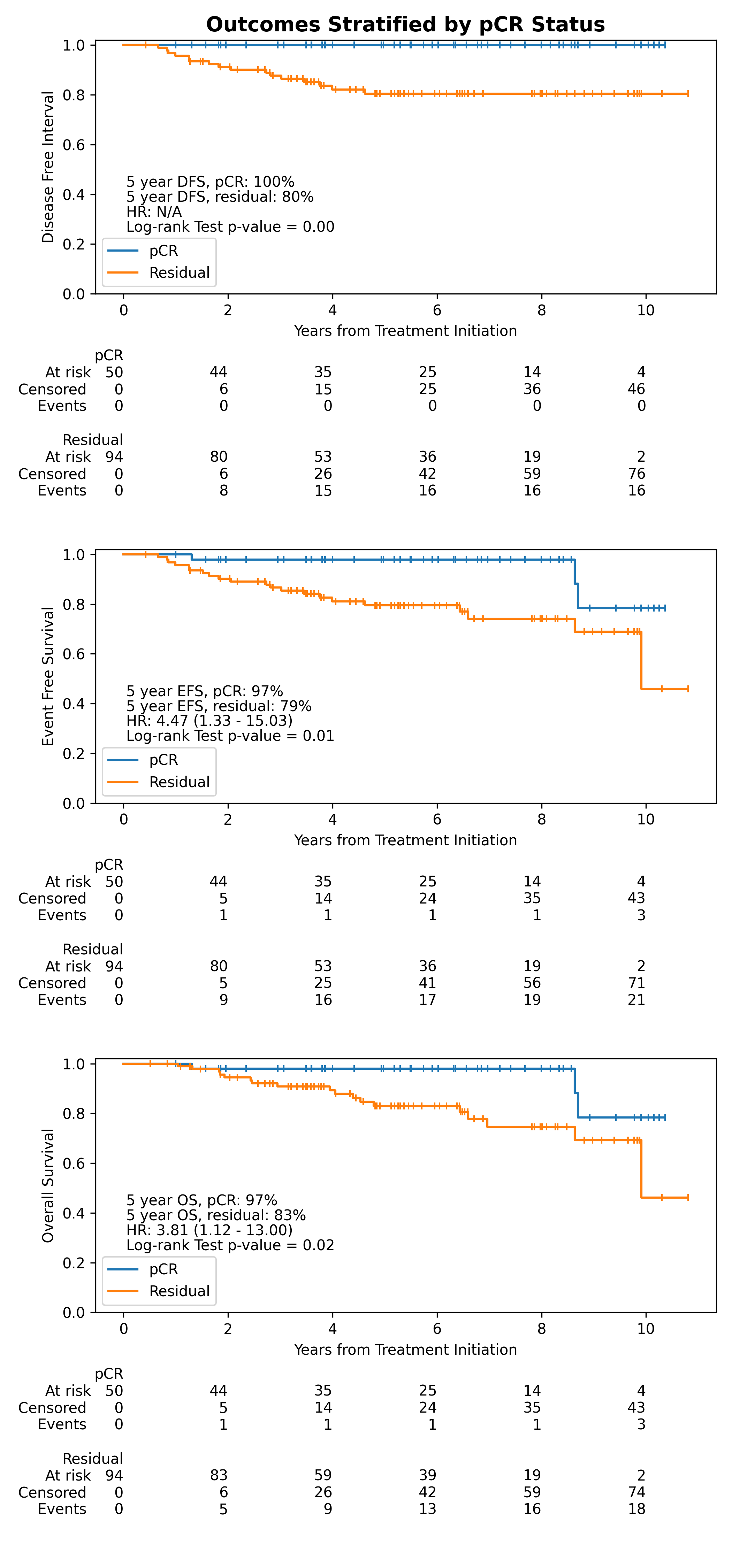


**Supplemental Figure 5. Disease Free Interval, Event Free Survival, and Overall Survival, Stratified by Pathologic Complete Response Status.**


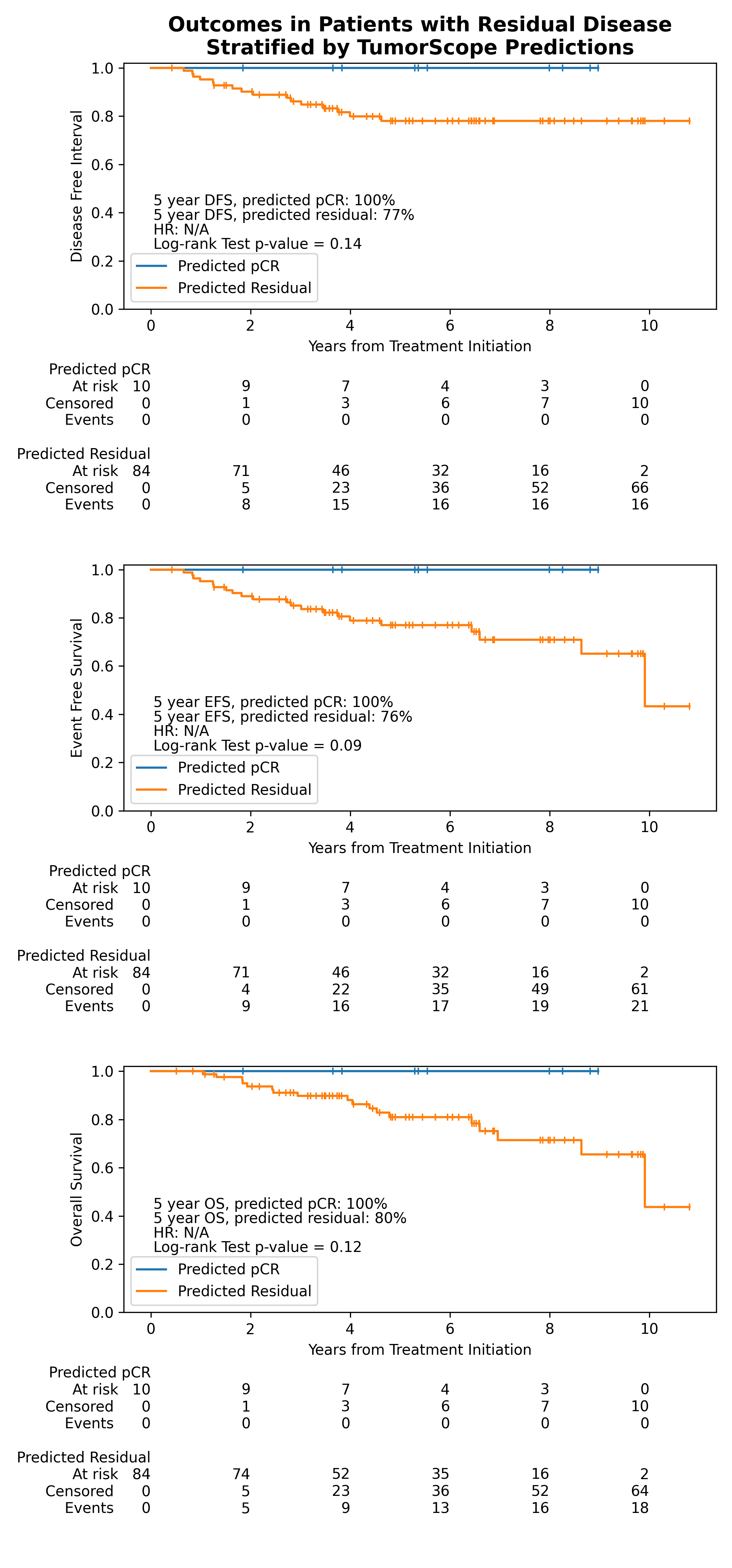


**Supplemental Figure 6. Disease Free Interval, Event Free Survival, and Overall Survival in Cases with Residual Disease, Stratified by Model Response Prediction.**

**Supplemental Table 3. Neoadjuvant Treatment Regimens by Number of Cases Treated.** Abbreviations: T = paclitaxel or docetaxel. A = doxorubicin. C = cyclophosphamide. Cb = carboplatin. H = trastuzumab. P = pertuzumab.

| **Regimen** | **n Cases** |
| --- | --- |
| T-AC / AC-T | 78 |
| TCbHP | 17 |
| TCbH | 15 |
| THP-AC | 11 |
| TH-AC / AC-TH | 7 |
| TC | 5 |
| AC-TCb | 3 |
| THP | 3 |
| AC | 1 |
| TCbH-AC | 1 |
| weekly T | 1 |
| nabT-AC | 1 |
| T-AC-gemcitabine | 1 |

**Supplemental Table 4. Pathologic Data for Patients with Predicted Pathologic Complete Response but Residual Disease.**

| **Case** | **Subtype** | **Residual Breast Tumor** | **Cellularity (%)** | **Nodes Positive** | **Largest Nodal Metastasis (mm)** |
| --- | --- | --- | --- | --- | --- |
| 1 | TNBC | No | 0 | 2 of 13 | 2.5 |
| 2 | HR+/HER2- | No | 0 | 1 of 5 | 2.1 |
| 3 | TNBC | Yes | N/A^a^ | 0 of 3 | 0 |
| 4 | HR+/HER2- | Yes | N/A^b^ | 0 of 5 | 0 |
| 5 | HR-/HER2+ | Yes | <3 | 0 of 17 | 0 |
| 6 | TNBC | Yes | <1 | 0 of 19 | 0 |
| 7 | TNBC | No | 0 | 6 of 11 | 16 |
| 8 | HR+/HER2- | Yes | 5 | 0 of 3 | 0 |
| 9 | TNBC | Yes | 20 | 3 of 20 | 13 |
| 10 | HR-/HER2+ | No | 0 | 2 of 24 | 0.5 |

^a^ Single microscopic focus of residual disease, cellularity not reported.

^b^1 cm focus of residual disease, cellularity not reported

**Supplemental Table 5. Outcome Metrics, Overall and Select Subgroups for Prediction of Pathologic Complete Response when Modeling Response Without Dose Reductions.**

|  | **n** | **n pCR** | **Accuracy (95% CI)** | **Sensitivity (95% CI)** | **Specificity (95% CI)** |
| --- | --- | --- | --- | --- | --- |
| **Overall** | 144 | 54 | 86.8 (80.2 - 91.9) | 88.0 (75.7 - 95.5) | 86.2 (77.5 - 92.4) |
| **HR+/HER2-** | 36 | 7 | 86.1 (70.5 - 95.3) | 80.0 (28.4 - 99.5) | 87.1 (70.2 - 96.4) |
| **HER2+** | 49 | 23 | 87.8 (75.2 - 95.4) | 91.3 (72.0 - 98.9) | 84.6 (65.1 - 95.6) |
| **TNBC** | 59 | 24 | 86.4 (75.0 - 94.0) | 86.4 (65.1 - 97.1) | 86.5 (71.2 - 95.5) |
| **Anthracycline-containing regimens** | 103 | 40 | 84.5 (76.0 - 90.9) | 88.2 (72.5 - 96.7) | 82.6 (71.6 - 90.7) |
| **Anthracycline-free regimens** | 41 | 14 | 92.7 (80.1 - 98.5) | 87.5 (61.7 - 98.4) | 96.0 (79.6 - 99.9) |

**Supplemental Table 6. Mean Absolute Error in Volumetric Predictions**

| **Timepoint** | **Number of Scans** | **Mean Absolute Error (95% CI)** | **Median Absolute Error (Range)** |
| --- | --- | --- | --- |
| **Overall** | 411 | 9.62 (7.94 - 11.31) | 1.23 (0.00 - 126.35) |
| **Pre-treatment** | 144 | 2.66 (1.19 - 4.13) | 0.41 (0.00 - 73.92) |
| **Inter-regimen** | 144 | 17.79 (13.66 - 21.92) | 8.47 (0.00 - 126.35) |
| **Post-treatment** | 123 | 9.61 (7.22 - 12.00) | 3.77 (0.00 - 72.51) |
